# Supplementary material for: Polyketide synthase gene clusters in the ericoid mycorrhizal fungus Oidiodendron maius: genome-wide prediction, expression profiling, and functional characterization of knockout mutants
Source: Front Plant Sci. 2026 Sep 16;17:1924430. doi: 10.3389/fpls.2026.1924430 (PMC13623903; doi:10.3389/fpls.2026.1924430)
Supplement: Supplementary file 1 [file SupplementaryFile1.zip › Supplementary Materials/Supplementary Folder 1.zip!/Oidma1_AssemblyScaffolds_Repeatmasked/knownclusterblast/cluster1/jgi.p_Oidma1_108363_mibig_hits.html]

| MIBiG Protein | Description | MIBiG Cluster | MiBiG Product | % ID | % Coverage | BLAST Score | E-value |
| --- | --- | --- | --- | --- | --- | --- | --- |
| BAV32149.1 | cytochrome\_P450\_monooxygenase | BGC0001373 | Sordarin\_biosynthetic\_gene\_cluster | 33 | 100.766283525 | 276 | 7.2e-74 |
| AAK33073.1 | cytochrome\_P450 | BGC0001278 | Nivalenol\_/\_deoxynivalenol\_/\_3-acetyldeoxynivalenol\_/ | 34 | 93.6781609195 | 269 | 6.7e-72 |
| AAK33083.1 | putative\_cytochrome\_P450 | BGC0001277 | Nivalenol\_/\_deoxynivalenol\_/\_3-acetyldeoxynivalenol\_/ | 33 | 97.1264367816 | 264 | 3.7e-70 |
| AAK53584.1 | trichodiene\_oxygenase | BGC0000931 | Trichothecene\_biosynthetic\_gene\_cluster | 33 | 97.1264367816 | 263 | 4.8e-70 |
| AAC49958.1 | cytochrome\_P450 | BGC0001279\_c2 | Deoxysambucinol\_/\_sambucinol\_/\_roridin\_E\_biosynthetic\_gene\_cluster | 29 | 99.6168582375 | 252 | 1.1e-66 |
| AAK53577.1 | trichodiene\_oxygenase | BGC0000930 | Trichothecene\_biosynthetic\_gene\_cluster | 32 | 97.1264367816 | 250 | 3.2e-66 |
| AAS89997.1 | CypA | BGC0000007 | Aflatoxin\_biosynthetic\_gene\_cluster | 32 | 101.724137931 | 238 | 1.7e-62 |
| Bcin12g06400.1 |  | BGC0000631 | Botrydial\_biosynthetic\_gene\_cluster | 31 | 100.957854406 | 233 | 5.4e-61 |
| ADM79460.1 | P450\_protein | BGC0001266 | Grayanic\_acid\_biosynthetic\_gene\_cluster | 29 | 109.38697318 | 223 | 7.2e-58 |
| BAV32152.1 | cytochrome\_P450\_monooxygenase | BGC0001373 | Sordarin\_biosynthetic\_gene\_cluster | 29 | 101.724137931 | 206 | 9.2e-53 |
| AAS90045.1 | CypA | BGC0000009 | Aflatoxin\_biosynthetic\_gene\_cluster | 33 | 68.3908045977 | 186 | 7.5e-47 |
| XP\_002384857.1 | cytochrome\_P450,\_putative | BGC0001234\_c2 | 4,4'-piperazine-2,5-diyldimethyl-bis-phenol\_biosynthetic\_gene | 25 | 99.8084291188 | 140 | 4.7e-33 |
| BAD29972.1 | P450\_monooxygenase\_1 | BGC0000676 | Aphidicolin\_biosynthetic\_gene\_cluster | 26 | 91.9540229885 | 127 | 5.4e-29 |
| BAE56591.1 |  | BGC0001123 | Aspirochlorine\_biosynthetic\_gene\_cluster | 26 | 90.8045977011 | 121 | 2.3e-27 |
| EFY94501.1 | benzoate\_4-monooxygenase\_cytochrome\_P450 | BGC0000337 | Destruxins\_biosynthetic\_gene\_cluster | 25 | 91.3793103448 | 121 | 3.9e-27 |
| CDM36724.1 | Cytochrome\_P450 | BGC0001360 | Mycophenolic\_acid\_biosynthetic\_gene\_cluster | 24 | 96.9348659004 | 112 | 1.4e-24 |
| AAM18769.1 | AflL | BGC0000011 | Aflatoxin/sterigmatocystin\_biosynthetic\_gene\_cluster | 26 | 90.9961685824 | 108 | 2.6e-23 |
| AAS90059.1 | VerB | BGC0000009 | Aflatoxin\_biosynthetic\_gene\_cluster | 26 | 92.1455938697 | 107 | 5.8e-23 |
| AAC49200.1 | putative\_p450\_monooxygenase | BGC0000152 | Sterigmatocystin\_biosynthetic\_gene\_cluster | 27 | 90.2298850575 | 106 | 7.6e-23 |
| AAS90079.1 | VerB | BGC0000010 | Aflatoxin\_biosynthetic\_gene\_cluster | 25 | 92.1455938697 | 102 | 1.9e-21 |
| AAS90033.1 | VerB | BGC0000008 | Aflatoxin\_biosynthetic\_gene\_cluster | 25 | 92.1455938697 | 102 | 1.9e-21 |
| AAS90102.1 | VerB | BGC0000006 | Aflatoxin\_biosynthetic\_gene\_cluster | 25 | 92.1455938697 | 102 | 1.9e-21 |
| AAS90011.1 | VerB | BGC0000007 | Aflatoxin\_biosynthetic\_gene\_cluster | 25 | 91.5708812261 | 101 | 2.4e-21 |
| BAE71326.1 | desaturase | BGC0000004 | Aflatoxin\_biosynthetic\_gene\_cluster | 25 | 92.1455938697 | 99 | 1.2e-20 |
| NP\_199610.1 | cytochrome\_P450\_705A5 | BGC0000670 | Thalianol\_biosynthetic\_gene\_cluster | 21 | 79.8850574713 | 86 | 1.4e-16 |
| EED17999.1 | benzoate\_4-monooxygenase\_cytochrome\_P450,\_putative | BGC0000154 | Stipitatic\_acid\_biosynthetic\_gene\_cluster | 30 | 40.6130268199 | 86 | 1.4e-16 |
| AEE83587.1 | cytochrome\_P450,\_family\_705,\_subfamily\_A,\_polypeptide\_2 | BGC0001313 | Arabidiol\_/\_Baruol\_biosynthetic\_gene\_cluster. | 22 | 92.5287356322 | 85 | 1.8e-16 |
| AFT91368.1 | hypothetical\_protein | BGC0000338 | Echinocandin\_B\_biosynthetic\_gene\_cluster | 25 | 91.5708812261 | 82 | 1.2e-15 |
| ACD75398.1 | cytochrome\_P450\_monooxygenase\_CYP52-M1 | BGC0001274\_c5 | Non-acetylated\_open-chain\_sophorolipid\_/\_diacetylated\_lactonic | 30 | 32.7586206897 | 76 | 1.1e-13 |
| BAD86796.1 | putative\_P450 | BGC0000286 | Viguiepinol\_biosynthetic\_gene\_cluster | 27 | 52.1072796935 | 76 | 1.4e-13 |
| CAQ18837.1 | putative\_cytochrome\_P450 | BGC0000954 | Ajudazol\_biosynthetic\_gene\_cluster | 31 | 30.4597701149 | 74 | 3.2e-13 |
| AEE83590.1 | cytochrome\_P450,\_family\_705,\_subfamily\_A,\_polypeptide\_4 | BGC0001313 | Arabidiol\_/\_Baruol\_biosynthetic\_gene\_cluster. | 27 | 31.4176245211 | 72 | 2.1e-12 |
| AEE83588.1 | cytochrome\_P450,\_family\_705,\_subfamily\_A,\_polypeptide\_3 | BGC0001313 | Arabidiol\_/\_Baruol\_biosynthetic\_gene\_cluster. | 28 | 32.7586206897 | 72 | 2.1e-12 |
| NP\_199072.1 | cytochrome\_P450,\_family\_705,\_subfamily\_A,\_polypeptide\_12 | BGC0000669 | Marneral\_biosynthetic\_gene\_cluster | 34 | 24.9042145594 | 71 | 2.7e-12 |
| AEE83585.1 | cytochrome\_P450,\_family\_705,\_subfamily\_A,\_polypeptide\_1 | BGC0001313 | Arabidiol\_/\_Baruol\_biosynthetic\_gene\_cluster. | 25 | 34.0996168582 | 67 | 6.6e-11 |
| EER93097.1 | hypothetical\_protein | BGC0000798 | Dhurrin\_biosynthetic\_gene\_cluster | 27 | 32.9501915709 | 64 | 5.6e-10 |
| AAN74818.2 | Fum15p | BGC0000062 | Fumonisin\_biosynthetic\_gene\_cluster | 22 | 66.2835249042 | 62 | 2.1e-09 |
| Manes.12G133500 |  | BGC0001318 | Linamarin\_/\_Lotaustralin\_biosynthetic\_gene\_cluster. | 25 | 32.5670498084 | 61 | 3.6e-09 |
| AGA37282.1 | P450\_monooxygenase | BGC0000819 | Paraherquamide\_biosynthetic\_gene\_cluster | 24 | 34.2911877395 | 57 | 4e-08 |
| chr3.CM0241.700.r2.m |  | BGC0001316 | Linamarin\_/\_Lotaustralin\_biosynthetic\_gene\_cluster. | 25 | 26.0536398467 | 54 | 3.4e-07 |
| CBF87243.1 | cytochrome\_P450,\_putative\_(Eurofung) | BGC0000680\_c2 | Austinol/dehydroaustinol,\_locus\_1\_biosynthetic\_gene\_cluster | 25 | 35.8237547893 | 52 | 1.3e-06 |
